# Hints on the Associated Use of Oil of Turpentine and Mercury

**Published:** 1829-06

**Authors:** John Wilson


### OIL OF TURPENTINE AND MERCURY.

*Hints on the associated Use of Oil of Turpentine and Mercury.* By John Wilson, m.d. r.n. (Communicated by Dr. Wm. Burnett.)

At page 37 of the "Memoirs of West Indian Fever," there is the following passage: "Like mercury, turpentine excites the vascular system primarily, and increases secretion secondarily, though not so steadily or powerfully; it acts, however, more suddenly, and it appears to dispose the body to be more readily impressed by mercury."

I now propose to detail a few cases in confirmation of the opinion expressed in the latter part of the preceding sentence, and to make some cursory remarks on the therapeutic agency of calomel and oil of turpentine in combination; in which I shall endeavour to show that the latter, under proper management, accelerates the action and increases the curative power of the former. When I speak of accelerating the action and increasing the curative power of mercury, I do not, of course, refer exclusively, or even principally, to one of the chief signs of that action, namely, ptyalism; but to excitement of the general capillary system, whether secreting or unsecreting, in which I believe its remedial power, at least in febrile disease, mainly to consist. Whether turpentine, when combined with calomel, possesses any influence beyond accelerating the action and increasing the power of mercury as a mercurial, is difficult to determine; but I am disposed to think that in certain cases it has a specific power of its own, increased by being associated with the specific power of mercury. To make my meaning more clear, I believe that the combination in question, like compound purgatives, has greater effect than the effects of the individual remedies taken together; and that the turpentine, in such cases, does not act simply as a non-cathartic stimulant given with a purge, which accelerates the action and increases the power of the purgative medicine, insomuch as it excites the lining membrane of the intestine, thereby alone quickening the purgative action and rendering it greater; but that it cooperates directly with the mercury in the remedial process, and contributes a part to the whole sanative effect.

Opium has generally been given with the calomel, but with the view chiefly of preventing its acting strongly on the mucous coat of the intestine: this it has generally accomplished, and in some instances has probably acted beneficially otherwise.

It often happens that, when we are anxious to excite the constitutional action of mercury, we find it difficult or unpracticable to do so, from the nature of the morbid action which we wish to subvert, or from some peculiarity in the organization of the patient, which, on the contrary, sometimes precipitates the mercurial action. The latter cause of difficulty is so inscrutable, that it may have led me to false conclusions in some of the cases in which I have used turpentine and calomel in combination; but, notwithstanding the deception to which that source of error may lead, I think myself justified in assuming that the conclusions which I have drawn are, upon the whole, just: and, if turpentine possess the power alleged, if it have simply the effect of accelerating mercurial action, it must be admitted to form a valuable auxiliary, at least, in combating some very serious forms of disease. In the graver forms of congestive fever, especially within the tropics, where we rest our principal hopes of success on mercurial action, and find ourselves baffled hour after hour in exciting that action, while we are persuaded that a few hours must seal the patient's fate, such an auxiliary would be hailed with feelings of gratification, known only to those who have been so circumstanced: those who, while giving dose after dose of calomel, have watched for some sign of its action as a signal of safety, and have despaired of seeing it.

It may be thought that I should have waited for more certain proofs of my opinions before I submitted them to the profession, and so I once thought myself; but I have few opportunities of putting them to the test of further experience at present; and, as I deem them of some importance, I think it better to have them tried on a larger field than that of single observation, and therefore offer them, uncertain as they are, believing that, in all cases where it is proper to excite mercurial action, they may be tried at least with perfect safety.

The turpentine is given in different doses in different cases; so managed, however, as not to act sensibly on the intestines, and not to produce more than a slight degree of strangury.

I shall only premise further that the cases which follow are of a chronic nature, though I see no reason to doubt but that similar results may be obtained in acute cases. I may add, indeed, that the practice was first suggested to me by a case of fever, where, after giving large doses of calomel without any perceptible effect, oil of turpentine was given as a purge, which was speedily followed by well-marked mercurial action, and eventual recovery in very hopeless circumstances.

Case I.—John Cooper, aged forty, boatswain's mate, 23d May, 1826, (at Bermuda.) Complains of headach, pain in the right side, shoulders, and back; the pulse is not much accelerated, and is of good strength; tongue white and moist; constipation, and want of appetite.—To take a purgative bolus of calomel and jalap.

24th.—Headach relieved, and there is less pain, excepting in the right hypochondrium and epigastric region. Medicine has acted fully. General appearance as yesterday. On examining the right hypochondrium, the liver is found considerably enlarged and hardened, and he complains of increased pain on handling. Says that he has suffered from the same complaint before.—V.S. ad ℥xx. followed by faintness and vomiting.

25th.—Complaint of side little changed, and he has considerable dyspnœa, without cough. Had a bad night, with startings and sense of impending suffocation, which forced him to sit up in bed. There is little change otherwise in the symptoms.—A large blister, embracing the right hypochondrium and part of the chest applied.

26th.—Blister has acted well, and he expresses himself as considerably relieved. There is not so much dyspnœa, and the sense of suffocation is less distressing: there is still, however, fixed pain in the hypochondrium, extending to the scapula, and impeding respiration; tongue clean; bowels open; little thirst, no appetite; skin natural.—Capiat mane Ol. Terebinth. ʒij., et vespere Subm. Hydrarg. gr. ij. in pil.

27th.—Report nearly as yesterday. Blistered surface discharging freely.—Let the turpentine be repeated in the morning, and the calomel pill in the evening, as yesterday.

28th.—Says that he is better, that he has slept well, and breathes more freely. Bowels open, with frequent micturition. Some return of appetite. Blistered surface nearly dried up.—Turpentine and calomel to be repeated as before.

29th.—Signs of mercurial action: gums swelled, and increased secretion of saliva. Continues to improve.

30th.—Considerable flow of saliva; and there is no complaint except a sense of confinement in the right side on deep inspiration, and that is much less than it was. From this date till the 8th of May he mended progressively, when he returned to duty free from complaint.

Case II.—James Whitehead, aged thirty-two years, (February 7th, 1827, at Port Royal,) was discharged to duty on the 18th of January, cured of tertian intermittent, which went off under the use of cinchona and bitters in large doses. To day he has had two smart attacks of periodic fever, one in the fore and the other in the afternoon, each paroxysm having lasted about three hours. At present free from fever.—A full dose of calomel and jalap.

8th.—Has continued free from fever, but complains of a good deal of pain in the shoulders and hypochondrium, with sense of weariness and weight about the shoulders. Has been frequently and fully purged in the night.

9th.—Had a slight attack yesterday forenoon, the three stages being passed through in two hours. Complains of hypochondria, and shoulders.—No medicine.

10th.—Has had two distinct paroxysms today, similar to those of the 7th, and one yesterday, but is at present free from fever. Complains of weakness, pain in the sides, with slight cough, and sense of weariness. The countenance is sallow. There is tenderness in the hypochondriac and epigastric regions. He has not been exposed again, so far as is known, to the cause of intermittent fever: it is therefore probable that the recurrence of the complaint depends on visceral obstruction, especially of the liver.—Submuriat. Hydrarg. gr. iv., Opii gr. i. fiat pil., to be taken at bedtime.

11th.—Much as last night.—To take two drachms of oil of turpentine in mucilage, and the calomel and opium pill to be taken again in the evening.

12th.—Has had no return of fever. Gums swollen and tender, breath has the mercurial fetor.—Turpentine in the morning, and bolus in the evening, as yesterday.

13th.—Copious salivation.—No medicine, excepting a dose of salts, and an astringent gargle.

15th.—No return of fever. Cough, complaint of hypochondria, and shoulders, gone. Copious salivation still.

18th.—Free from complaint. Duty.

Case III.—In the following case turpentine was not given with the calomel: it is introduced for the purpose of comparing it with the next in subsequent order, in which calomel was associated with turpentine, and showing the advantage of the combination.

John Sheppard, aged thirty, (30th March, 1826, at Bermuda,) is affected similarly to John Grave,* (whose case is detailed at page 9 of the Journal;) but in this case the symptoms of neuralgia are more severe than in the former. The pain comes on in an instant, and in an instant the eye becomes inflamed, and the tears run down the cheek. Independent of his own account, it is obvious that his suffering is very severe. It is referred to the notch or hole through which the frontal twig of the ophthalmic branch of the fifth pair of nerves passes from the orbit to the forehead; and from that point exactly he describes the pain as shooting up, and ramifying over the forehead; sometimes it darts around the ridge forming the edge of the orbit. It is most acute from seven o'clock till twelve in the forenoon, but returns occasionally at irregular periods. The circulation, digestion, and other functions, are not perceptibly affected. After free purging, he was ordered, on the evening of the second day, as in the case of Grane, Submuriat. Hydrarg., Pulv. Antimon., āā gr. x.; Opii gr. x. M.

April 2d.—The pain is less acute than yesterday. Medicine repeated.

3d.—Pain as yesterday. Medicine repeated as yesterday and the day before.

4th.—Pain much less acute. Medicine repeated.

5th.—Little pain left, but he complains of numbness in the parts affected. Medicine continued.

6th.—Report as yesterday. Medicine as before. It deserves remark, that there is neither constipation nor gastric derangement of any kind; no appearance of salivation.

7th.—No complaint. Omit the medicine.

8th.—Disease does not threaten to return. Discharged to duty.

Case IV.—John Sheppard (21st of January, 1827, at sea,) has continued free from complaint since the 8th of April, 1826, when he was cured of neuralgia: it has now, however, returned, affecting the same place in the same manner, though not quite so severely as before.—To take an ipecacuan emetic.

* I regret that I have mislaid the notes of this case.

22d.—No change. A full dose of calomel and jalap; to be followed by salts and senna, if necessary to effective purging.

23d.—The bowels have been thoroughly emptied; and the pulse, tongue, and skin give no indication of disease, but the complaint in the orbit and forehead remain unchanged. No medicine.

24th.—Report as yesterday. In the evening to take the following bolus: Submuriat. Hydrarg. gr. v., Opii gr. i. M.

25th.—Little change since yesterday. Bolus to be repeated in the evening.

26th.—Says the pain is less acute. To take two drachms of oil of turpentine in mucilage now, and the bolus at night.

27th.—Has had very little pain since yesterday. Gums swollen, pulse accelerated; skin rather hot; some heat of urine. Turpentine as yesterday.—Evening: Copious salivation; pain gone. No medicine.

28th.—Continues free from complaint, except of his mouth, which is very sore, with abundant discharge of saliva. No medicine.

31st.—The salivation continues copious. No return of the original complaint; general headach. A saline purge.

February 1st.—Headach removed. Report in other respects as before.

4th.—Salivary discharge more moderate. No complaint.

7th.—Mouth well. No return of the neuralgic pain; no complaint. Discharged to duty.

In this case, when twenty grains of calomel were taken, abundant salivation ensued: in the former case, sixty grains were taken without inducing ptyalism. The two cases occurred in the same person, and as nearly as possible in the same circumstances: the same disease, the same mode of life, and apparently the same general condition of the body. There may have been some imperceptible or unperceived agency acting on the system, which rendered it less susceptible of the mercurial action at one period, and more susceptible at the other; but I think no closer evidence can be obtained in such cases, and it appears fair to infer, in the last, that the turpentine accelerated the action and increased the power of the mercury.

Case V.—Anne Charles, (6th May, 1828,) the mother of many children, and fifty years of age, presents the following symptoms: Extreme emaciation and weakness; pulse hurried, weak and irregular, occasionally intermittent; tongue florid, shining, and smooth; skin hard, dry, and cold, especially on the lower extremities, though she complains at times of deep-seated, burning heat; tenderness in epigastrio on pressure; no appetite; bowels irregular, on the whole costive, with ill-conditioned discharges; subject, during the last month, to fainting fits, with slight convulsive movements of the limbs; there is deep-seated pain about the sacrum and hips, darting down the back of the thighs, so severe as almost entirely to prevent sleep, and force her to scream. From these pains she is never free entirely; but they are much more acute at one time than another, and most distressing on alternate days. She has been ill about six months, and from herself and occasional medical attendant I gathered the following particulars: She was first affected with symptoms of catarrhal fever, for which she was bled, and took the usual remedies, till the acute symptoms gave way. Instead of recovering strength, however, she continued poorly, with flying pains, which at length fixed where they now are: she had little appetite; was distressed with flatulence and other hysteric symptoms; could not sleep, and continued to waste. She took aperients, cordials, and bitters, then diaphoretics; employed frictions and blisters; and had, on one occasion, taken daily, for a fortnight, a pill containing, besides compound colocynth extract and rhubarb, a grain and a half of calomel. In spite of every thing, she continued getting worse till the 6th of May, when I found her in the condition already stated; a condition so unpromising, that I had little hope from treatment of any kind.

What had been done appeared judicious, though ineffective; and what to attempt further, I was at a loss to determine. In what way the pains of the limbs, of which she now alone complained, were connected with the gastric affection? and what that gastric affection was? I did not know. But one thing pressed itself strongly on the attention, namely, the dry, cold, inanimate state of the surface, especially in the lower extremities, showing the want of adequate circulation there, and the probability of dangerous, deep-seated congestion. This state I determined to make my chief practical guide, and accordingly (hot baths could not be obtained,) ordered a pill, consisting of ten grains of calomel and a grain and a half of opium, at night; and, the following morning, two drachms of oil of turpentine in mucilage; with an occasional draught, containing carbonate of ammonia and compound tincture of cinnamon.

It would be tedious to give daily details of the symptoms, and I shall therefore only state that, when she had taken five pills and as many of the turpentine draughts, a most unexpected and gratifying change appeared: the pains were much abated, the surface was warm and soft, the appetite considerable, the pulse of good strength; and the gums were tumid and tender, without salivation. The tongue was white, but not loaded; the bowels acted regularly, and she menstruated. There being then some heat of urine, the turpentine was omitted for a day, the pill being continued.

When she had taken the medicines eleven days, the mouth being sore, she objected to taking more; which, as the disease seemed to be removed, were not urged upon her. She then took considerable doses of the sulphate of quinine for ten days; and, three months after the commencement of the course, she walked twelve miles in a day, to visit a daughter.

The foregoing cases are introduced principally for the purpose of showing that oil of turpentine accelerates the action and increases the power of mercury as a mercurial; but I think they besides furnish hints, and point to conclusions of a different, though not less interesting, nature, as I have before suggested. It would lead me beyond the limits of a paper like this, to comment on each of them; but I beg to offer a remark or two on the cases of John Sheppard and Anne Charles, all of them, I believe, certainly the two first, instances of neuralgic disease; and I regret that I have mislaid the notes of two other cases of similar import.

In the case of Anne Charles, calomel had been given, in combination with aperients, to a large amount; then it was afterwards given with turpentine. In the first instance it had no sensible, at any rate it had no beneficial, effect. There was no perceptible difference in the condition of the patient, except the difference produced by the uninterrupted progress of the disease. It appears, therefore, fair to conclude that the turpentine did more than dispose the body for the speedy action of the mercury; that it cooperated essentially with it, that it contributed a constituent, though unknown, part to the curative effect.

In the cases of John Sheppard, it is true, the disease yielded under the use of mercury, without being associated with turpentine, in the first instance; and perhaps nothing more can properly be deduced from them, than that the operation of the one was quickened by that of the other. It is to be observed, however, that, after the cure effected under the mercury without turpentine, the disease returned at the end of eight months; whereas, when I last saw the subject, twenty months had elapsed without any return of symptoms.

There is little affinity between the following case and any of the preceding ones: its history is unsatisfactory and its nature obscure, and I am not satisfied regarding the part which turpentine acted in its cure; yet I am tempted to insert it, because it is, in some respects, uncommon and interesting.

John Shaw, aged twenty-four, (7th November, 1826, at Port Royal,) complained three days ago of headach, for which he was purged; says that there is severe pain both in the occiput and sinciput, though there is no appearance of disease, the pulse, tongue, and skin appearing healthy.—A dose of calomel and jalap.

8th.—Says he is no better: the breath is offensive, and there are other appearances of diseased digestion. An emetic given.

9th.—Still complains as before, the general appearance being as before. V.S. ad ℥xx., which induced faintness, &c. To take a full dose of calomel and jalap.—Evening: There being no change, a blister was applied to the nape of the neck.

10th.—Says that the pain in the hind head is gone, but that the forehead continues as it was, and that the pain now affects the eyeballs and sockets. The bowels have not been satisfactorily purged. To take a draught of salts and senna every two hours, till there be full catharsis.

11th.—The bowels are now thoroughly emptied, and he says he is much better.

12th.—Continues better: walks about, and takes food.

13th.—Report as yesterday.

14th.—Complains again of pain across the forehead and affecting the eyes. The left temple to be cupped and scarified.

15th.—No change. A blister to the temples. There is still no apparent constitutional derangement.

17th.—He became delirious in the night, and refused to stay in bed; and in the morning was partially comatose, being roused with difficulty, and answering questions indistinctly. Pulse fifty-eight, of moderate strength; skin cooler than natural; tendency to coldness in the extremities. Head shaved, and the scalp covered with a blister. To take a scruple of calomel.—Evening: No change. Calomel repeated in scruple dose.

18th.—Pulse has risen to seventy, and acquired strength; skin warmer; he is more easily roused, but there is still comatose tendency. Calomel repeated.—Noon: a drachm of oil of turpentine. —Evening: The blister has acted pretty well. Calomel repeated.

19th.—Frequent and tolerably abundant discharges of urine, and he appears rather better. Calomel as before.—Noon: half an ounce of oil of turpentine.—Evening: calomel repeated.

20th.—Little change since last night. Urine, which is tinged with blood, passed frequently and unconsciously; numerous stools; gums tumid and tender. Calomel repeated.

In the afternoon, having returned to Port Royal, he was sent to hospital, passing urine and feces unconsciously, and appearing to labour under hopeless oppression of the brain. That the brain was oppressed to a certain extent, can scarcely be doubted; but it is now pretty clear that the oppression consisted in vascular congestion merely; for, soon after going to the hospital, on the breaking out of a copious salivation, the alarming symptoms suddenly disappeared: he became sensible; natural appetite, and the other functions of health were restored; and in a fortnight afterwards he returned to duty.

In this case, it will be admitted that life was in danger; and it will also be admitted, I think, that it was saved through the twofold agency of excitement being communicated to the vascular tissue, and the consequent evacuation. It may not be admitted that the means employed were the most appropriate; but, whether that be admitted or denied, the mercury was the direct agent, acting in its twofold capacity, of the patient's recovery. In what manner or measure the oil of turpentine contributed to the successful issue, is uncertain, but I think it did contribute. When calomel had been given in large quantity without any perceptible effect, and when, from the impaired condition of the nervous organs, and general torpor, it was doubtful whether it entered the system, the turpentine was soon found to have mingled with the blood, and acted on the secernants. Having done so, may it not be supposed to have excited the absorbents to take up, and the blood-vessels to move the calomel onwards. If it did so, it would clearly be of great importance in a case like this.

It is in such cases of acute disease that turpentine promises to be a valuable auxiliary to the curative means now in use, which are too often inadequate under the best management, and therefore want the aid of others. In deep and dangerous congestion, affecting organs immediately necessary to life, arising from a state of the cerebral system little understood, could we be assured of the specific action of mercury, we should be less apprehensive of the issue: hence any thing which had the power of precipitating that action would be an important acquisition. In febrile disease, it is to such cases that turpentine should be chiefly applied; and to such cases, indeed, I believe it ought to be limited. It ought not to be administered where there is general inflammatory action, unless it be given as a purgative remedy, and then it does not appear to be an appropriate purge. Like calomel, it ought to be given when there is diminution, not increase, of vascular action.

